# A Threat- and Efficacy-Based Framework to Understand Confidence in Vaccines among the Public Health Workforce

**DOI:** 10.3390/vaccines1020077

**Published:** 2013-04-08

**Authors:** Daniel J. Barnett, Nicole A. Errett, Lainie Rutkow

**Affiliations:** 1 Department of Environmental Health Sciences, Johns Hopkins Bloomberg School of Public Health, 615 North Wolfe Street, Room E7036, Baltimore, MD 21205, USA; 2 Department of Health Policy and Management, Johns Hopkins Bloomberg School of Public Health, 624 North Broadway, Room 513, Baltimore, MD 21205, USA; E-Mails: nerrett@jhsph.edu (N.A.E.); hrutkow@jhsph.edu (L.R.)

**Keywords:** preparedness, confidence, self-efficacy, vaccines, extended parallel process model, legal protections, law

## Abstract

The Extended Parallel Process Model (EPPM) is an established threat- and efficacy-based behavioral framework for understanding health behaviors in the face of uncertain risk. A growing body of research has applied this model to understand these behaviors among the public health workforce. In this manuscript, we aim to explore the application of this framework to the public health workforce, with a novel focus on their confidence in vaccines and perceptions of vaccine injury compensation mechanisms. We characterize specific connections between EPPM’s threat and efficacy dimensions and relevant vaccine policy frameworks and highlight how these connections can usefully inform training interventions for public health workers to enhance their confidence in these vaccine policy measures.

## 1. Introduction

Health providers’ personal decisions not to receive influenza vaccination present a major ongoing public health challenge, with significant implications for morbidity and mortality, medical workforce capacity and healthcare system costs [1]. Low rates of seasonal influenza vaccination among physicians in the U.S. and Israel, for example, reflect the international scope of physicians’ own non-adherence to this critical preventive behavior [1,2].

A growing body of research has examined perceptual barriers impeding health providers’ self-adherence to influenza vaccination guidelines. To date, such identified barriers have included a lack of information about novel and seasonal influenza vaccines, the risk of influenza infection, the role of the healthcare worker in transmission and vaccine safety and efficacy [3,4,5,6]. Motivational factors have included the desire to protect self and family through vaccination [5].

Of note, modifying factors for providers’ own influenza vaccination behavior appear to be similar for both seasonal and pandemic influenza contexts [6,7,8]. Indeed, these factors resonate with findings from studies that we and other researchers have conducted on pandemic flu response willingness-related attitudes among healthcare providers, including physicians [9,10].

Senior public health organizational and regulatory bodies (e.g., Centers for Disease Control and Prevention, World Health Organization, national health ministries) are leading purveyors of influenza vaccination practice guidance for physicians. Accordingly, the effectiveness of these public health authorities in mitigating influenza disease burden hinges substantially on healthcare providers’ adherence to the vaccine guidelines that these agencies deliver—for themselves and their patients.

Public health department-based providers represent a critical component of the frontlines of vaccine delivery and are at the heart of the public health emergency preparedness system [11,12]. Despite the importance of public health workers in this context, the research literature has yet to sufficiently examine how this provider cohort’s perceptions toward vaccination and vaccine injury compensation may influence their vaccine-related behaviors for themselves and the communities they serve. Recent research suggests that the Extended Parallel Process Model (EPPM), an established threat- and efficacy-based behavioral framework for understanding health behaviors in the face of uncertain risk, could be usefully applied to understand such vaccine-related perceptions among the public health workforce [13,14,15,16]. In this article, we aim to explore the potential utility of explicitly applying the EPPM to examine public health providers’ confidence in vaccines and vaccine-injury compensation mechanisms.

Specifically, we will explore these EPPM-based implications in the following contexts: (1) National Vaccine Injury Compensation Program; (2) Public Readiness and Emergency Preparedness Act (PREP Act) for Pandemic Influenza Medical Countermeasures Utilization Protocol & Decision Tools; and (3) mandatory vaccination policies.

Each of these three contexts involves a unique legal infrastructure with specific components that may impact public health workers’ confidence in vaccines. In particular, we focus on the compensation mechanisms that the government has established for individuals and families that experience vaccine-related adverse events. Public health workers’ awareness and understanding of these systems may influence their perceptions of threat and efficacy and, thus, their willingness to receive a vaccination. 

## 2. The Extended Parallel Process Model and Its Application to Confidence in Vaccines

Introduced by Witte in 1992, the EPPM has the potential to cast light on how the public health workforce can be encouraged to participate in influenza vaccination. The model posits that for a message to induce behavior change, it must simultaneously convey the constructs of threat and efficacy. The construct of threat has two components: severity or the idea that the threat is significant enough to warrant action; and susceptibility or the idea that the person initiating the behavior may be affected by the threat. The construct of efficacy also has two components: self-efficacy or the idea of confidence that the person is able to perform the behavior; and response efficacy or the idea that the behavior change will achieve the intended impact. If the message induces both perceived threat and perceived efficacy in the recipient, the message may be accepted [16]. If the message induces high levels of perceived threat, but does not promote perceived efficacy, fear will be elicited and the recipient will undergo what is known as “fear control”. In this situation, the message may be rejected or defensively avoided [17]. Importantly, self-efficacy or the confidence component of efficacy, has been identified as the component most associated with actual behavior adaptation [18].

The EPPM has been both qualitatively and quantitatively applied to or suggested for a variety of preventive health behaviors, including low income mothers’ access to preventive dental care for their children, contraceptive use among women, colonoscopy screening, hearing loss prevention and smoking cessation intentions [19,20,21,22,23,24]. The relative importance of confidence has been affirmed in predicting preventive behavior engagement in some of this work. For example, among smokers with low readiness to quit, threat and efficacy were both seen as important predictors of intention to engage in smoking cessation behavior. However, among those with high readiness to quit, efficacy was most important in this prediction [24].

The foundation for the use of this model to understand and promote preventive behaviors toward influenza has recently been developed. Notably, in a study utilizing EPPM-framed forewarning messages to promote preventive behaviors, such as vaccination for H5N1 influenza, perceived threat was associated with fear arousal, which was less positively related to behavioral intention than perceived efficacy [25]. These findings underscore the role of confidence in determining a person’s potential decision to get vaccinated relative to an individual’s perceived threat. In a qualitative study of African American seniors employing the EPPM, issues related to accessing an influenza vaccine dominated the discussion related to efficacy. This population, which receives lower levels of vaccination compared to the general population, identified accessibility, affordability, negative consequences of vaccine (e.g., side effects), physician recommendation and efficacy of vaccination as emergent themes associated with efficacy [26]. In a study of individuals aged 65 and older, while messages developed under the EPPM framework were not shown to significantly increase intention to receive vaccination, the use of the framework in message design did significantly positively influence perceptions of risk and efficacy [27]. 

## 3. Laws and Policies of Relevance to Public Health Providers’ Confidence in Vaccines

Here, we describe in greater detail the three aforementioned law and policy contexts germane to confidence in vaccines among public health providers: (1) National Vaccine Injury Compensation Program; (2) Public Readiness and Emergency Preparedness Act (PREP Act) for Pandemic Influenza Medical Countermeasures Utilization Protocol & Decision Tools; and (3) mandatory vaccination policies.

### 3.1. National Vaccine Injury Compensation Program

Each year, millions of people in the United States are vaccinated to prevent the morbidity and mortality associated with various infectious diseases. Although the effectiveness and general safety of vaccines have been repeatedly demonstrated, they are not without risk. On rare occasions, some individuals experience side effects, including seizures and anaphylactic shock after receiving a vaccination [28]. Known as vaccine-related adverse events, these responses raise important questions, especially for vaccinations that are recommended by the government. One of the most salient questions to emerge is what, if any, compensation should individuals or families receive after experiencing a vaccine-related adverse event?

In the mid-1980s, Congress tackled this issue with the drafting and passage of legislation to establish the National Vaccine Injury Compensation Program (VICP) [29]. The VICP is a government-run program, housed in the U.S. Department of Health and Human Services (HHS). It offers compensation to individuals who qualify, based on the vaccine they received and the nature of the adverse event they experienced. Numerous vaccines are covered by the VICP, including diphtheria, tetanus, pertussis, measles, mumps, rubella and polio. Importantly for public health workers, the VICP covers both Haemophilus influenzae and trivalent influenza vaccines. A publicly available table lists all vaccines and adverse events covered by the program (e.g., anaphylactic shock within four hours of receiving a tetanus vaccination) [30]. To be eligible, claims for compensation must be filed within three years of the initial occurrence of the adverse event. In general, the claim should concern a vaccine and an adverse event that occurred within a timeframe listed on the table. Once a claim is filed, a physician within HHS makes an initial determination about whether the medical information in the claim meets the qualifications for compensation. This recommendation is then shared with a special master tasked with making compensation decisions for the VICP. Compensation may include coverage for medical expenses, attorney’s fees, lost earning capacity and death benefits for survivors [31]. The VICP provides some liability protections for individuals who administer vaccines that are covered by the program.

### 3.2. Public Readiness and Emergency Preparedness Act

While the VICP’s protections are available to both children and adults, it was intended to cover vaccinations routinely offered to children. The VICP does not offer compensation for vaccine-related adverse events associated with emergency countermeasures, such as vaccines used to treat H1N1 pandemic influenza or smallpox. To address this gap, Congress established the Countermeasures Injury Compensation Program (CICP) with the passage of the Public Readiness and Emergency Preparedness (PREP) Act in 2006 [32]. 

Like the VICP, the CICP is housed in the U.S. Department of Health and Human Services. It provides compensation to individuals who experienced an adverse event related to a countermeasure they received that was covered by the CICP [33]. Compensation may cover medical expenses, lost income and death benefits for survivors. Unlike the VICP, the CICP only covers countermeasures that are listed in a declaration from the Secretary of the Department of Health and Human Services. This declaration must specify the disease that poses or is likely to pose a public health emergency, the countermeasure that is covered to treat the disease, the period for which the declaration will remain in effect and any population or geographic limitations for administration of the countermeasure [34]. Claims for compensation under the CICP must be filed within one year of the administration of the countermeasure. Since the PREP Act’s inception, the Secretary has issued declarations that covered vaccine countermeasures for smallpox, acute radiation syndrome, anthrax and botulism, as well as pandemic influenza, including the H1N1 and H5N1 strains [35]. The PREP Act includes liability protections for individuals who participate in the development and administration of covered countermeasures [36].

### 3.3. Mandatory Vaccination of Healthcare Workers

Over a century ago, the U.S. Supreme Court established that states can require individuals to receive vaccinations to protect and promote the public’s health [37]. All states, for example, have vaccination requirements that children must meet before they can attend school, with exceptions for individuals with medical contraindications and—in some states—exemptions for those with religious and philosophical objections. These requirements are associated with coverage rates of approximately 95% for children entering kindergarten in the U.S. [38]. In recent years, state and local governments and private employers have grappled with requiring mandatory seasonal influenza vaccinations for healthcare workers who have routine patient contact. These policies, which have been implemented by healthcare institutions in nearly half of the states, contain exemptions for healthcare workers who have medical contraindications [39]. Some also allow healthcare workers to sign a declination statement and take additional precautions (e.g., wearing a mask) if they refuse the vaccination. The policies typically do not mention compensation for vaccine-related adverse events. Should an adverse event occur, compensation would potentially be available from the VICP for seasonal influenza vaccinations. In addition, compensation could be available for pandemic influenza vaccinations from the CICP if the Secretary of Health and Human Services issued a declaration to cover the countermeasure.

Mandatory seasonal influenza policies have faced legal challenges from healthcare workers who, in some cases, argue that they contravene collective bargaining agreements. Such lawsuits, which tend to be highly publicized, can undermine confidence in vaccines, as they may raise questions for the general public about the safety and effectiveness of vaccines [40].

## 4. Role of Law in Behavior Change

PREP, VICP and mandatory vaccination policies could potentially serve as effect modifiers toward public health workers’ confidence in vaccines, with attendant implications for vaccine uptake rates among this vital health provider cohort. In that vein, the law has long been recognized as a policy tool with a demonstrated ability to impact behavior. Laws can cause numerous changes—to environments, to product availability, to the legality of certain practices—that may influence individuals’ behaviors. While the implementation of a new law may be associated with behavior change, it may also occur due to the amendment, repeal or expiration of an existing law or to other factors entirely. Two brief examples, from tobacco control and motor vehicle safety, illustrate the law’s potential to influence behavior change.

Tobacco control researchers have repeatedly found that the law is a powerful component of smoking cessation and prevention efforts. The implementation of legal measures, such as the presence of tobacco taxes, which raise the price of cigarettes, has been associated with individuals’ increased intention to quit smoking, as well as their decreased purchase of cigarettes. A systematic review of tobacco taxes and smoking behavior concluded that a robust evidence base demonstrates that increasing the price of cigarettes through tobacco taxes is associated with the reduction of smoking, particularly among young people [41]. Several studies have examined clean indoor air laws through the lens of behavior change theories. These studies have similarly concluded that certain theories, such as the theory of planned behavior, can help to explain the interplay between individuals’ intentions to quit smoking and public policies designed to limit tobacco use. For example, in a study grounded in a conceptual model based on the theory of planned behavior, Middlestadt and colleagues found that current smokers who lived in a city with a smoke-free air law and who had high intentions to quit had greater odds of engaging in quitting behaviors than those who had lower intentions to quit and lived in cities without smoke-free air laws [42]. Recently, some researchers have modeled how possible legal approaches—such as a ban on menthol cigarettes—might influence the behavior of current smokers. O’Connor and colleagues found that this type of ban would potentially be associated with quit attempts by approximately one-third of individuals who currently smoke mentholated cigarettes [43].

Studies have repeatedly confirmed the important role that laws have played in reducing the number of annual traffic fatalities in the United States, particularly due to their impact on seatbelt use behavior [44]. For example, Chaudhary and colleagues determined that primary enforcement seatbelt laws are associated with increases in both daytime and nighttime seatbelt use [45]. Primary enforcement laws allow police to issue citations for failure to wear a seatbelt; secondary enforcement laws require the presence of another traffic violation before police can give a citation for lack of seat belt use. In an analysis that considered multiple federal and state data sources, Carpenter and Stehr found that laws requiring the use of seatbelts—and particularly those laws that allowed for primary enforcement when seatbelt laws were violated—were associated with statistically significant increases in the use of seatbelts by adolescents [46]. In addition, they found that primary enforcement laws for seatbelts significantly decreased traffic-related deaths within this age group. Laws intended to reduce drinking and driving, especially among young people, have also been associated with statistically significant decreases in motor vehicle fatalities [47,48]. These types of results suggest that laws can influence behavior changes and thus contribute to improved motor vehicle safety.

Against this backdrop of law and its relationship to behavior change, EPPM can serve as a useful lens to gauge the extent to which VICP, PREP and mandatory vaccination policies may serve as effect modifiers toward vaccine confidence. In addition, the application of EPPM to this area may assist in the design of relevant training approaches for public health workers’ awareness of and confidence in available protections. Here, the recent public health preparedness literature provides relevant insights. Specifically, EPPM has recently been applied to several cohorts involved in the public health workforce to understand and improve their willingness to respond to a variety of emergencies in the all-hazards disaster spectrum, including pandemic influenza. 

Notably, the self-efficacy confidence construct was consistently identified as the leading overall predictor of willingness to respond to a pandemic influenza emergency among all cohorts studied [14,49,50,51]. Indeed, EPPM-based research has found that efficacy outweighs threat as a modifier of public health workers’ response willingness toward infectious disease and other crisis scenarios [14]. The implications of these EPPM-related findings are described below.

## 5. Implications of EPPM for Vaccine Policy Contexts and Confidence

EPPM-based research to date on emergency response willingness points to the applicability of a threat- and efficacy-based behavioral model for future applied research aimed at understanding the interplay between legal protections and confidence in vaccines. 

A closer examination of how EPPM’s threat and efficacy components apply to the three vaccine contexts discussed above illustrates EPPM’s considerable potential as a conceptual framework for vaccine confidence-boosting initiatives against these policy backdrops. In the case of the VICP, for example, EPPM’s threat dimension applies to public health workers’ concerns about experiencing a vaccine-related adverse event. According to EPPM principles, instilling awareness of this threat dimension (*i.e*., potential, though rare, adverse events) can motivate public health workers to seek additional information about the protections that the VICP affords and how to access these protections should an adverse event occur. Meanwhile, EPPM’s efficacy dimension in this context applies to public health workers’ confidence in operational implementation of VICP as needed: in the case of self-efficacy, these workers can gain confidence in their knowledge of the VICP protections available to them and they can simultaneously increase their confidence by understanding the requisite protocols to access VICP protections. Under PREP, the elements of EPPM would apply in a similar way to the CICP, with the only difference being that the CICP applies to a more circumscribed set of vaccine-related countermeasures. Finally, in the context of mandatory vaccination, the threat dimension of EPPM reflects public health workers’ potential concerns that they may serve as vectors of vaccine-preventable illness to their own families and to the vulnerable patient populations they serve. With regard to EPPM’s efficacy domain in the case of mandatory vaccination policies, self-efficacy refers to public health workers’ confidence in their comprehensive understanding of the mandatory policy, a thorough knowledge of where to go to receive vaccinations and a clear awareness of whom to contact if they encounter a vaccine-related adverse event. Further, in the context of mandatory vaccination policies, EPPM’s response efficacy dimension would reflect these workers’ confidence that mandatory vaccination actually works to mitigate the likelihood of disease transmission.

Importantly, EPPM-centered training is the common operational thread that binds these conceptual understandings with practical implementation strategies that can boost confidence in vaccination amidst the three vaccine-policy backdrops described above. EPPM-centered training efforts could enhance public health workers’ confidence in vaccines through threat- and efficacy-building elements. Specifically, the threat elements of such training should address the respective vaccine policy threat dimensions noted above, as the EPPM indicates that threat awareness can have a positive motivational effect toward adoption of desired behaviors (in this case, vaccination against these respective policy backdrops). Meanwhile, such EPPM-centered training activities should include a particularly strong emphasis on efficacy-building training measures surrounding these vaccine policies, as efficacy has been found to outweigh threat as a modifier of public health workers’ response behaviors in the face of perceived risk [14]. The efficacy component of such EPPM-centered trainings would need to focus on: (1) boosting public health workers’ confidence in their understanding of and knowledge of how to access respective protections accompanying these measures (*i.e*., self-efficacy per the EPPM); and (2) instilling a clear awareness that the protections afforded are meaningful and substantive and would be fully implemented (*i.e.*, response efficacy per EPPM). The importance of explicit awareness—and confidence in—workplace protective measures is bolstered by the research finding that perceived safety at work is a highly significant modifier of public health workers’ willingness to fulfill role expectations in a variety of disaster contexts [14]. 

## 6. Conclusions

The role of legal protections in boosting confidence in vaccines has become increasingly relevant in an environment where mandatory vaccination for public health workers is a contentious policy issue. Further, EPPM-centered research could assist policy-makers, emergency planners and others to better understand root determinants of the influence of legal protections on vaccine uptake decisions among public health providers. Additionally, as noted above, the EPPM can be used to inform training interventions to enhance public health workers’ awareness of relevant vaccination policies and confidence in them.

Future EPPM-based research on policy and practice can thus provide a vital evidence base for enhanced understanding of how public health workers’ confidence in vaccines is modified by relevant protective laws, through the lens of an established threat- and efficacy-centered behavioral model. This type of research can focus not only on public health workers, but also on other health provider cohorts on the frontlines of community vaccination efforts.

## References

[1] Caban-Martinez A.J., Lee D.J., Davila E.P., LeBlanc W.G., Arheart K.L., McCollister K.E., Christ S.L., Clarke T., Fleming L.E. (2010). Sustained low influenza vaccination rates in US healthcare workers. Prev. Med..

[2] Habib S., Rishpon S., Rubin L. (2000). Influenza vaccination among healthcare workers. Isr. Med. Assoc. J..

[3] DeSante J.E., Caplan A., Shofer F., Behrman A.J. (2010). Physician attitudes towards influenza immunization and vaccine mandates. Vaccine.

[4] Douville L.E., Myers A., Jackson M.A., Lantos J.D. (2010). Healthcare worker knowledge, attitudes, and beliefs regarding mandatory influenza vaccination. Arch. Pediatr. Adolesc. Med..

[5] Hollmeyer H.G., Hayden F., Poland G., Buchholz U. (2009). Influenza vaccination of healthcare workers in hospitals—A review of studies on attitudes and predictors. Vaccine.

[6] Rehmani R., Memon J.I. (2010). Knowledge, Attitudes and beliefs regarding influenza vaccination among healthcare workers in a saudi hospital. Vaccine.

[7] Vírseda S., Restrepo M.A., Arranz E., Magán-Tapia P., Fernández-Ruiz M., de la Cámara A.G., Aguado J.M., López-Medrano F. (2010). Seasonal and pandemic A (H1N1) 2009 influenza vaccination coverage and attitudes among health-care workers in a Spanish university hospital. Vaccine.

[8] Esteves-Jaramillo A., Omer S.B., Gonzalez-Diaz E., Salmon D.A., Hixson B., Navarro F., Kawa-Karasik S., Frew P., Morfin-Otero R., Rodriguez-Noriega E. (2009). Acceptance of a vaccine against novel influenza a (H1N1) virus among healthcare workers in two major cities in Mexico. Arch. Med. Res..

[9] Irvin C.B., Cindrich L., Patterson W., Southall A. (2008). Survey of hospital healthcare personnel response during a potential avian influenza pandemic: Will they come to work?. Prehosp. Disaster Med..

[10] Garrett A.L., Park Y.S., Redlener I. (2009). Mitigating absenteeism in hospital workers during a pandemic. Disaster Med. Public Health Prep..

[11] Institute of Medicine of the National Academies, USA (2002). The Future of the Public’s Health in the 21st Century.

[12] Institute of Medicine of the National Academies, USA (2008). Research Priorities in Emergency Preparedness and Response: A Letter Report.

[13] Seib K., Barnett D.J., Weiss P.S., Omer S.B. (2012). Vaccine-related standard of care and willingness to respond to public health emergencies: A cross-sectional survey of california vaccine providers. Vaccine.

[14] Barnett D.J., Thompson C.B., Errett N.A., Semon N.L., Anderson M.K., Ferrell J.L., Freiheit J.M., Hudson R., Koch M.M., McKee M. (2012). Determinants of emergency response willingness in the local public health workforce by jurisdictional and scenario patterns: A cross-sectional survey. BMC Public Health.

[15] Barnett D.J., Balicer R.D., Thompson C.B., Storey J.D., Omer S.B., Semon N.L., Bayer S., Cheek L.V., Gately K.W., Lanza K.M. (2009). Assessment of local public health workers’ willingness to respond to pandemic influenza through application of the extended parallel process model. PLoS One.

[16] Witte K. (1992). Putting the fear back into fear appeals: The extended parallel process model. Commun. Monogr..

[17] Witte K. (1994). Fear control and danger control: A Test of the extended parallel process model. Commun. Monogr..

[18] Bandura A. (1977). Self-efficacy: Toward a unifying theory of behavioral change. Psychol. Rev..

[19] Askelson N.M., Chi D.L., Momany E., Kuthy R., Ortiz C., Hanson J.H., Damiano P. (2012). Encouraging early preventive dental visits for preschool-aged children enrolled in medicaid: Using the extended parallel process model to conduct formative research. J. Public Health Dent..

[20] Campo S., Askelson N.M., Spies E.L., Losch M. (2012). Ambivalence, communication and past use: understanding what influences women’s intentions to use contraceptives. Psychol.Health Med..

[21] Pengchit W., Walters S.T., Simmons R.G., Kohlmann W., Burt R.W., Schwartz M.D., Kinney A.Y. (2011). Motivation-based intervention to promote colonoscopy screening: an integration of a fear management model and motivational interviewing. J. Health Psychol..

[22] Kotowski M.R., Smith S.W., Johnstone P.M., Pritt E. (2011). Using the extended parallel process model to create and evaluate the effectiveness of brochures to reduce the risk for noise-induced hearing loss in college students. Noise Health.

[23] Murray-Johnson L., Witte K., Patel D., Orrego V., Zuckerman C., Maxfield A.M., Thimons E.D. (2004). Using the extended parallel process model to prevent noise-induced hearing loss among coal miners in Appalachia. Health Educ. Behav..

[24] Wong N.C., Cappella J.N. (2009). Antismoking threat and efficacy appeals: Effects on smoking cessation intentions for smokers with low and high readiness to quit. J. Appl. Commun. Res..

[25] Siu W. (2008). Extended parallel process model and H5N1 influenza virus. Psychol. Rep..

[26] Cameron K.A., Rintamaki L.S., Kamanda-Kosseh M., Noskin G.A., Baker D.W., Makoul G. (2009). Using theoretical constructs to identify key issues for targeted message design: African American Seniors’ perceptions about influenza and influenza vaccination. Health Commun..

[27] Prati F., Pietrantoni L., Zani B. (2012). Influenza vaccination: the persuasiveness of messages among people aged 65 years and older. Health Commun..

[28] Health Resources and Services Administration (2011). What You Need to Know about the National Vaccine Injury Compensation Program (VICP).

[29] Rutkow L., Maggy B., Zablotsky J., Oliver T.R. (2007). Balancing consumer and industry interests in public health: The National Vaccine Injury Compensation Program and its influence during the last two decades. Penn. State Law Rev..

[30] Health Resources and Services Administration, U.S. Department of Health and Human Services Vaccine Injury Table. http://www.hrsa.gov/vaccinecompensation/vaccinetable.html/.

[31] (2012). United States Code, Title 42, Section 300aa-15(a).

[32] (2005). Public Readiness and Emergency Preparedness (PREP), Act in 2006. Public Law Number 109-148.

[33] Health Resources and Services Administration, Countermeasures Injury Compensation Program. http://www.hrsa.gov/gethealthcare/conditions/countermeasurescomp/index.html/.

[34] (2012). United States Code, Title 42, section 247d-6d(b).

[35] (2010). Pandemic Influenza Vaccines—Amendment. Federal Register. 75, 10,268.

[36] Crawford K.S., Axelrad J. (2012). Legislative modifications to tort liability: the unintended consequence of public health and bioterrorism threats. Creighton Law Rev..

[37] (1905). Jacobson v. Commonwealth of Massachusetts, 197 U.S. 11.

[38] Centers for Disease Control and Prevention (2012). Vaccination coverage among children in Kindergarten-United States, 2011-21 School Year. MMWR Morb. Mortal Wkly. Rep..

[39] Stewart A.M., Rosenbaum S. (2010). Vaccinating the health-care workforce: State law *vs.* institutional requirements. Public Health Rep..

[40] Parmet W.E. (2010). Pandemic vaccines—The legal landscape. N. Engl. J. Med..

[41] Bader P., Boisclair D., Ferrence R. (2011). Effects of tobacco taxation and pricing on smoking behavior in high risk populations: A knowledge synthesis. Int. J. Environ. Res. Public Health..

[42] Middlestadt S.E., Macy J.T., Seo D.C., Jay S.J., Kolbe L.J. (2012). The combined effect of behavioral intention and exposure to a smoke-free air law on taking measures to quit smoking. Health Promot. Pract..

[43] O’Connor R.J., Bansal-Travers M., Carter L.P., Cummings K.M. (2012). What would menthol smokers do if menthol cigarettes were banned? Behavioral intentions and simulated demand. Addiction.

[44] Strine T.W., Beck L., Bolen J., Okoro C., Li C. (2012). Potential moderating role of seat belt law on the relationship between seat belt use and adverse health behavior. Am. J. Health Behav..

[45] Chaudhary N.K., Tison J., Casanova T. (2010). The Effects of maine’s change to primary seat belt law on seat belt use and public perception and awareness. Traffic Inj. Prev..

[46] Carpenter C.S., Stehr M. (2008). The effects of mandatory seatbelt laws on seatbelt use, motor vehicle fatalities, and crash-related injuries among youth. J. Health Econ..

[47] Traynor T.L. (2009). The impact of state level behavioral regulations on traffic fatality rates. J. Safety Res..

[48] Fell J.C., Fisher D.A., Voas R.B., Blackman K., Tippetts A.S. (2009). The impact of underage driving laws on alcohol-related fatal crashes of young drivers. Alcohol Clin. Exp. Res..

[49] Balicer R.D., Barnett D.J., Thompson C.B., Hsu E.B., Catlett C.L., Watson C.M., Semon N.L., Gwon H.S., Links J.M. (2010). Characterizing hospital workers’ willingness to report to duty in an influenza pandemic through threat- and efficacy-based assessment. BMC Public Health.

[50] Errett N.A., Barnett D.J., Thompson C.B., Tosatto R., Austin B., Schaffzin S., Ansari A., Semon N.L., Balicer R.D., Links J.M. (2013). Assessment of medical reserve corps volunteers emergency response willingness using a threat- and efficacy-based model. Biosecur. Bioterror..

[51] Barnett D.J., Levine R., Thompson C.B., Wijetunge G.U., Oliver A.L., Bentley M.A., Neubert P.D., Pirrallo R.G., Links J.M., Balicer R.D. (2010). Emergency medical services workers willingness to respond to pandemic influenza using a threat- and efficacy-based assessment framework. PLoS One.

